# Electroacupuncture Treatment on Sarcopenia in Patients Undergoing Maintenance Haemodialysis: An Effective Therapy

**DOI:** 10.1002/jcsm.70345

**Published:** 2026-07-19

**Authors:** Ruoxin Chen, Qinqin Qian, Bin Wang, Ruoyang Chen, Liuping Zhang, Hui Jin, Jun Zhu, Zuolin Li, Jianyun Gao, Hong Liu

**Affiliations:** ^1^ Institute of Nephrology, Zhongda Hospital, School of Medicine Southeast University Nanjing Jiangsu China; ^2^ Institute of Acupuncture, Zhongda Hospital, School of Medicine Southeast University Nanjing Jiangsu China; ^3^ Institute of Nutrition, Zhongda Hospital, School of Medicine Southeast University Nanjing Jiangsu China; ^4^ Institute of Nephrology Taizhou Fourth People's Hospital Taizhou Jiangsu China

**Keywords:** electroacupuncture treatment, maintenance haemodialysis, sarcopenia, serum metabolomics

## Abstract

**Background:**

Electroacupuncture (EA) treatment has been utilized for recovery from neuromuscular‐related diseases and may play a significant role in the treatment of sarcopenia. This interventional, randomized controlled clinical study aims to explore the efficacy of EA treatment in maintenance haemodialysis (MHD) patients with sarcopenia.

**Methods:**

Thirty‐six participants with sarcopenia undergoing MHD were randomly divided into the control group and the EA group. The participants in the EA group received a total of 24 treatments, each lasting 30 min, and were administered three times per week. Participants in the control group were instructed to continue their current lifestyle and treatment plans. The assessments were conducted at baseline and after 8 weeks. Statistical analysis was performed using two‐way analysis of covariance (ANCOVA) adjusted according to gender and baseline values. Repeated measures analysis of variance (ANOVA) was used to assess EA effects, reporting main effects and the time × group interaction with partial eta squared (η^2^ *p*) effect sizes. The primary outcome was 6‐m gait speed; the secondary outcomes were skeletal muscle mass index (SMI) and handgrip strength. Fasting blood samples were collected, and serum metabolomics using the liquid chromatography–mass spectrometry method was employed to reveal metabolic changes.

**Results:**

One participant from the EA group dropped out, and 35 participants were included in the analysis, aged (59.06 ± 11.69) years, including 22 men and 13 women. After intervention, the 6‐m gait speed of the EA group increased (Δ = 0.10 ± 0.08; *p* < 0.001), whereas that of the control group decreased (Δ = −0.06 ± 0.09; *p* = 0.018). The handgrip strength of the EA group increased (Δ = 0.68 ± 0.98; *p* = 0.011), whereas that of the control group decreased (Δ = −0.76 ± 1.19; *p* = 0.015). The SMI in the EA group increased (Δ = 0.19 ± 0.22; *p* = 0.003), although there was no significant difference in the control group. No serious adverse events were observed during the EA treatment. The results of serum metabolomics indicated that a total of 127 differentially expressed metabolites were identified (*p* < 0.05, VIP > 1), including 35 up‐regulated metabolites and 92 down‐regulated metabolites. KEGG pathway enrichment analysis showed that glycerophospholipid metabolism, linoleic acid metabolism and other pathways related to lipid metabolism were significantly changed.

**Conclusions:**

EA treatment was an effective therapy for sarcopenia in patients undergoing MHD. Its therapeutic effect may be related to the positive regulation of systemic metabolism (including amino acid and lipid profiles).

## 1 Introduction

In recent years, the incidence of end‐stage kidney disease (ESKD) has been increasing year by year, with poor prognosis and high cost. Maintenance haemodialysis (MHD) is the main alternative treatment for ESKD, and about 83.8% of ESKD patients choose MHD [1]. Sarcopenia is a progressive and systemic degenerative syndrome characterized by decreased skeletal muscle mass and muscle function [2]. Our group reported that the prevalence of sarcopenia in MHD patients in our hospital was 28.17% [3], which was similar to the results of a previous meta‐analysis [4]. As a common complication of MHD patients, sarcopenia seriously endangers human health and increases the global economic burden [5, 6]. Therefore, targeted prevention and treatment of sarcopenia in MHD patients are of great significance.

Currently, the clinical management of sarcopenia is in an exploratory phase. The prevalent approaches primarily involve lifestyle interventions, such as exercise and nutritional supplementation [7]. However, for certain sarcopenia patients, engaging in exercise or even daily physical activities can be challenging [8]. Electroacupuncture (EA), an extension of traditional acupuncture, employs low‐frequency micro‐currents to intensify acupoint stimulation [9]. Studies have shown that EA treatment can improve blood flow and oxygenation of skeletal muscle, which is helpful in the treatment of muscle fatigue and musculoskeletal diseases [10]. In addition, EA treatment can regulate proangiogenic processes and protein turnover in gastrocnemius muscle, which may delay muscle atrophy in ageing mice [11]. Some preliminary studies suggested that EA treatment may also be significant in treating sarcopenia [12, 13, 14]. Notably, translational research bridging preclinical EA studies to human MHD populations remains critically lacking, particularly regarding pathophysiological mechanisms specific to uraemic sarcopenia.

Moreover, as the largest endocrine organ in the human body, EA treatment may affect the metabolism of skeletal muscle [15]. Analysing metabolic differences in patient samples collected before and after EA treatment could provide insights into potential therapeutic mechanisms. Therefore, we hypothesized that EA treatment can effectively improve sarcopenia in patients undergoing MHD. Furthermore, using metabolomics analysis methods, the effects of EA treatment on the metabolites in the bodies of these patients were investigated to explore potential mechanism pathways.

## 2 Methods

### 2.1 Study Design

This study was a single‐centre, outcome assessor‐blinded, parallel randomized controlled trial. Patients who underwent regular haemodialysis at Zhongda Hospital were selected as study subjects. Trial recruitment began on 1 January 2024 and ended on 31 March 2024. Patients were randomly assigned to the EA group and the control group in a 1:1 ratio (https://www.randomizer.org). Designated physicians enrolled participants who met the inclusion criteria and grouped the participants according to the results of randomization via the website. Personnel who recorded and assessed the assignments were unaware of the group assignments, and personnel who performed data analyses had no role in the study design or clinical care.

This study was approved by the independent ethics committee (IEC) for clinical research of Zhongda Hospital Affiliated to Southeast University (batch number: 2023ZDSYLL371‐P01). The trial has been registered in the Chinese Clinical Trial Registry (registration number: ChiCTR2400081511). The research was conducted in accordance with the principles of the Declaration of Helsinki. Informed consent for the study was obtained from all subjects and/or their legal guardians by signing an informed consent form.

### 2.2 Diagnosis of Sarcopenia

According to the 2019 revised Asian Working Group for Sarcopenia (AWGS) criteria, cut‐off values for these diagnostic components of sarcopenia were as follows: (a) Low muscle strength was defined as dominant handgrip strength < 28 kg for men and < 18 kg for women. (b) Low muscle mass using BIA was defined as skeletal muscle index (SMI) < 7.0 kg/m^2^ for men and < 5.7 kg/m^2^ for women. (c) The 6‐m gait speed < 1.0 m/s was recommended for the evaluation of physical ability. Sarcopenia was defined as low muscle mass accompanied by either low muscle strength or low physical performance [16]. SMI, handgrip strength and 6‐m gait speed were all measured within 30 min after the completion of haemodialysis. For specific measurement details of each index, refer to the methods section of our team's previous article on sarcopenia [3, 17, 18].

### 2.3 Study Populations

Inclusion criteria were as follows: (a) age > 18 years; (b) stable haemodialysis for more than 3 months; (c) regular haemodialysis three times a week for 4 h each time; and (d) meeting the diagnostic criteria for sarcopenia. The exclusion criteria were as follows: (a) unable to cooperate with EA treatment; (b) presence of malignant tumours, severe heart failure (NYHA IV), acute severe infection in the past 3 months, severe metabolic diseases, decompensated chronic liver disease, haematological diseases, platelet count less than 100 × 10^9^/L, etc.; (c) inability to complete the handgrip strength test or the 6‐m gait speed; (d) implanted with electronic devices or metallic materials; (e) received any treatment for sarcopenia in the 3 months before study entry or were participating in other clinical trials; (f) presence of severe cognitive impairment or mental illness; (g) pregnancy or lactation; and (h) lack of complete data.

### 2.4 Withdrawal Criteria

(a) Specialist physicians are responsible for assessing the serious adverse reactions or other needs that occurred during the study and deciding whether to continue or stop the study. (b) Subjects unable to continue EA treatment for various reasons. (c) Subjects withdrew informed consent.

### 2.5 Termination Criterion

If serious adverse reactions or events occurred, it was not appropriate to continue the trial.

### 2.6 Sample Size Calculation

Based on previous reports in the literature, the 6‐min walk distance (a measure of physical capacity) was used as the primary outcome measure [19]. The standard deviation (SD) of the expression of the experimental group and the control group was 94.73. The test power was set as 0.9, and the Type I probability error rate was 0.05 (two‐sided). According to N = (z_α_ + z_β_)^2^*2*σ^2^/δ^2^, PASS software was used to calculate the number of subjects in the test group and the control group required for this study, for a total of 32. Considering a dropout rate of 10%, the sample size used in the study was increased by 10% on an estimated basis. Thus, there were 18 participants each in the EA groups and control groups, for a total of 36 participants.

During the research design stage, no randomized controlled trials were found that investigated EA treatment on the core indicator of ‘6‐m gait speed’ in the population of MHD patients with sarcopenia. As a compromise, a literature on the effect of EA on physical function (measured by 6‐min walk distance) in MHD patients was referred to. This decision was made based on the following considerations: Both 6‐min walk distance and 6‐m gait speed are valid and reliable indicators for assessing the physical function and mobility of the elderly and patients with chronic diseases, and they are highly correlated [20, 21, 22].

### 2.7 Clinical Data

Baseline data, including the participant's family history, medical history and symptoms, complications and comorbidities, duration of dialysis and other basic information, were collected by an independent outcome assessor at the first visit. Information on the participants, instrument measures and other relevant data were collected at baseline and post‐treatment. Patient information was checked by nurses at the haemodialysis centre before dialysis, and fasting venous blood was collected after disinfection to detect the following indicators: haemoglobin, C‐reactive protein, albumin, serum creatinine, blood uric acid, total cholesterol, triglycerides and other biochemical indicators.

Blood samples for this study were standardized for storage and management in the biobank of Zhongda Hospital Affiliated with Southeast University. A dedicated biobank for the assessment of sarcopenia in patients undergoing MHD was established and organically integrated with specific topical research.

### 2.8 Interventions

#### 2.8.1 EA Group

**FIGURE 1:**
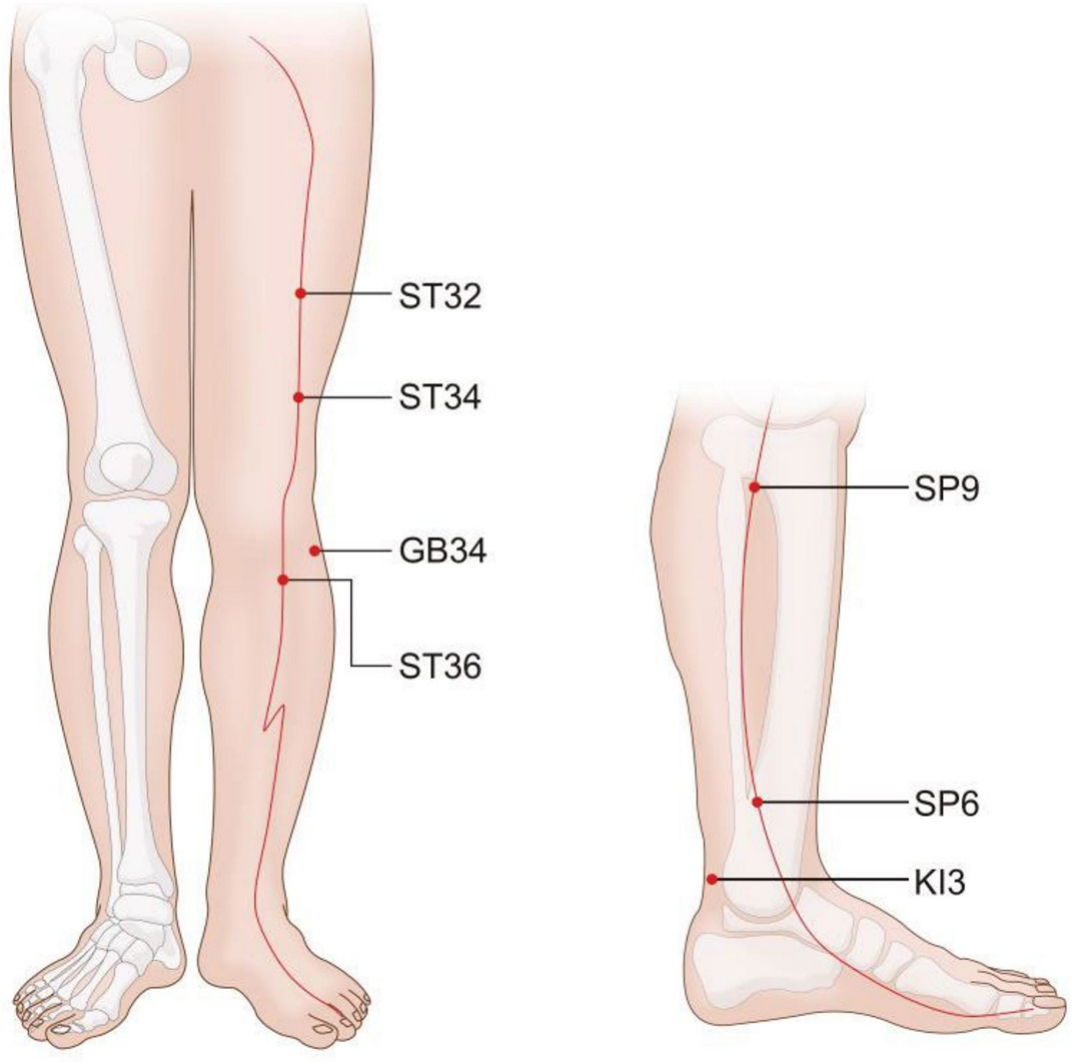
Schematic diagram of the acupoints.

After half an hour of haemodialysis, the patients were treated with EA while lying flat on the bed during stable dialysis. The patients were placed in the supine position, and Zusanli (ST36), Sanyinjiao (SP6), Futu (ST32), Yanglingquan (GB34), Yinlingquan (SP9), Taixi (KI3) and Liangqiu (ST34) were the selected acupoints. All the acupoints were selected bilaterally. The acupuncture points were identified according to the method of point location issued by the World Health Organization (WHO). The acupoint selection protocol used in this study was shown in Table 1, and a schematic diagram of acupoint selection was shown in Figure 1.

**TABLE 1:** Needling point and needle insertion procedures.

Needling point	Location	Depth and method
ST36 (Zusanli)	On the anterior aspect of the leg, on the line connecting ST35 with ST41, 3 B‐cun inferior to ST35	1–2 cun; perpendicular to the skin
SP6 (Sanyinjiao)	On the tibial aspect of the leg, posterior to the medial border of the tibia, 3 B‐cun superior to the prominence of the medial malleolus	1–2 cun; perpendicular to the skin
ST32 (Futu)	On the anterolateral aspect of the thigh, on the line connecting the lateral end of the base of the patella with the anterior superior iliac spine, 6 B‐cun superior to the base of the patella	1–2 cun; perpendicular to the skin
GB34 (Yanglingquan)	On the fibular aspect of the leg, in the depression anterior and distal to the head of the fibula	1–2 cun; perpendicular to the skin
SP9 (Yinlingquan)	On the tibial aspect of the leg, in the depression between the inferior border of the medial condyle of the tibia and the medial border of the tibia	1–2 cun; perpendicular to the skin
KI3 (Taixi)	On the posteromedial aspect of the ankle, in the depression between the prominence of the medial malleolus and the calcaneal tendon	0.5–0.8 cun; perpendicular to the skin
ST34 (Liangqiu)	On the anterolateral aspect of the thigh, on the line connecting the lateral end of the base of the patella with the anterior superior iliac spine, 6 B‐cun superior to the base of the patella	1–2 cun; perpendicular to the skin

The acupoints were routinely disinfected and treated with EA using a disposable sterile acupuncture needle with a diameter of 0.25 mm and a length of 40 mm (Andy brand, Guizhou, China). The depth of acupuncture was determined according to the thickness of the muscular layer, with straight needling at Taixi (0.5–0.8 cun) and other acupoints (1–2 cun). The ‘Deqi’ sensation was an evoked response of the patient and was acquired before the addition of electrical stimulation; this sensation includes pain, numbness, swelling or heaviness. After Deqi, the patients were treated with low‐frequency pulsed EA (XS‐998B04; Nanjing Xiaosong Medical Instrument Institute). Continuous waves, 2 Hz in frequency and 1–10 mA in intensity, were applied, and the patients' tolerance was considered. A total of 24 EA treatment sessions, 3 per week, were performed for a period of 8 weeks.

#### 2.8.2 Control Group

Participants in the control group did not receive EA treatment and continued to receive their normal usual treatment for the duration of the study.

### 2.9 Outcomes Measures

The following measures were measured at baseline and at Week 8. Primary outcome measures included 6‐m gait speed. The secondary outcome measures included SMI and handgrip strength.

### 2.10 Safety Evaluation

During the EA treatment, an acupuncturist and a nephrologist accompanied the patient throughout the process and monitored for any adverse reactions. Common adverse reactions include dizziness, needle sticking, needle bending, needle folding, skin infection at the acupuncture site, allergies, bleeding and pain. If the patient experiences any discomfort during the EA treatment, it is handled by professional doctors.

### 2.11 Metabolomics Study

Fasting cubital venous blood samples were collected from all patients at baseline and at the end of the experiment (Week 8) for metabolomics analysis. All collected blood samples were immediately sent to the Biobank of Zhongda Hospital affiliated with Southeast University, for centrifugation. The processed samples were stored in a −80°C refrigerator until they underwent metabolomics sequencing analysis. The methods included serum sample preparation, serum metabolomics based on gas chromatography–mass spectrometry and data analysis (shown in the Supporting Information). For the two groups of analysis, VIP (VIP > 1) and *p* values (*p* < 0.05, Student's *t*‐test) were extracted from the orthogonal partial least squares discriminant analysis (OPLS‐DA) results to determine the differential metabolites. The data were log transform (log2) and mean centring before OPLS‐DA. In order to avoid overfitting, a permutation test (200 permutations) was performed. Identified metabolites were annotated using KEGG Compound database, annotated metabolites were then mapped to KEGG Pathway database. The results of serum metabolomics were performed using the Metware Cloud, an online platform for data analysis (https://cloud.metware.cn) [23, 24, 25].

### 2.12 Statistical Analyses

Continuous variables were tested for normality using the Shapiro–Wilk test. Normally distributed continuous variables are presented as mean ± SD, and non‐normally distributed variables as median with interquartile range. Categorical variables are presented as counts and percentages and were compared using the chi‐square test or Fisher's exact test, as appropriate. To visually present the magnitude of changes before and after the intervention in each group, the two‐way analysis of covariance (ANCOVA) was used to compare the change scores from baseline to 8 weeks (Delta score, Δ), with sex and baseline value as covariates. To evaluate the effect of EA on the outcome measures, a 2 (time: baseline vs. 8 weeks) × 2 (group: EA vs. control) repeated measures analysis of variance (ANOVA) was performed. The main effects of time and group, as well as the time × group interaction, were reported. Effect sizes for the interaction were estimated using partial eta squared (η^2^ *p*). Statistical analysis was performed using SPSS software (Version 26.0, SPSS, Chicago, IL, USA). A *p* value of 0.05 was considered to be statistically significant.

## 3 Results

### 3.1 General Information

**FIGURE 2:**
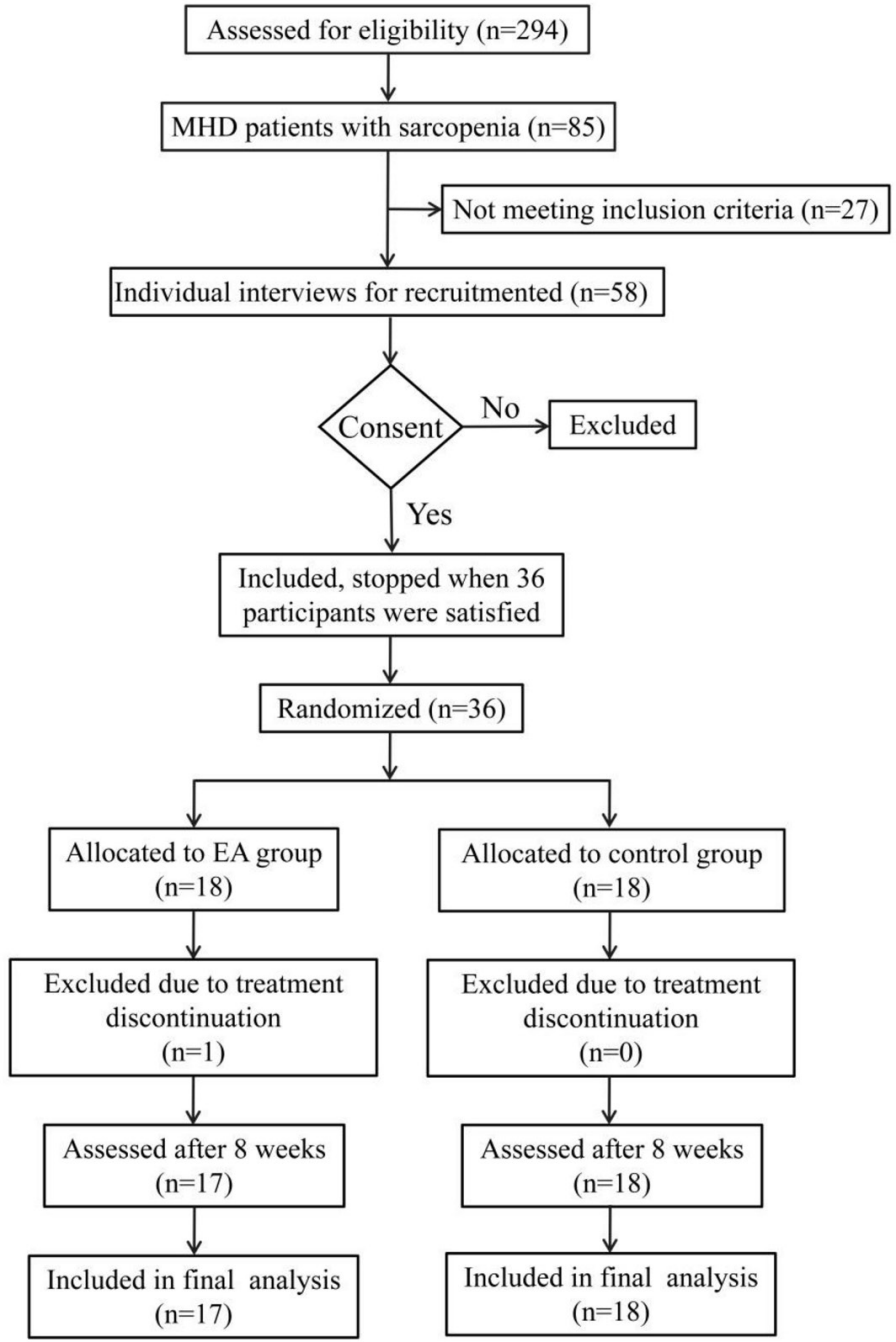
Flowchart.

The participant's characteristics, both stratified by sex and total, are presented in Table 2. A total of 36 patients were initially enrolled in the study and divided into the EA group (*n* = 18) and the control group (*n* = 18). During the EA treatment, one patient from the EA group withdrew due to surgical disease (Figure 2). Consequently, 35 patients were included in the analysis, with an average age of 59.06 ± 11.69 years, including 22 men and 13 women. The analysis results indicated that there was no significant difference in age, gender, BMI, underlying diseases and duration of dialysis between the EA group and control group (*p* > 0.05) (Table 2). Compared with women, more men smoked (*p =* 0.001) and drank alcohol (*p <* 0.001). Additionally, due to the natural differences in skeletal muscle between men and women, the diagnostic criteria for sarcopenia in men and women are also different. Compared with women, men had higher baseline handgrip strength (*p <* 0.001), SMI (*p <* 0.001) and 6‐m gait speed (*p =* 0.041).

**TABLE 2:** Characteristics of study participants.

Characteristic	CON‐men (*n* = 12)	EA‐men (*n* = 10)	CON‐women (*n* = 6)	EA‐women (*n* = 7)	CON (*n* = 18)	EA (*n* = 17)	*p*
Age (y)	59.92 ± 11.52	56.10 ± 14.22	65.00 ± 9.03	56.71 ± 10.11	61.61 ± 10.77	56.35 ± 12.33	0.188
BMI (kg/m^2^)	21.24 ± 1.31	20.30 ± 2.21	20.32 ± 1.91	20.54 ± 2.20	20.93 ± 1.54	20.40 ± 2.14	0.401
Tobacco use, *n* (%)	8	6	1	0	9	6	0.380^**a**^
Alcohol consumption, *n* (%)	11	7	2	0	13	7	0.064^**a**^
Hypertension, *n* (%)	11	9	4	7	16	16	0.581
Diabetes, *n* (%)	2	4	2	0	4	4	0.927
Total cholesterol (mmol/L)	4.20 ± 0.86	3.90 ± 0.84	3.47 ± 0.94	4.74 ± 1.60	3.96 ± 0.93	4.25 ± 1.24	0.441
Triglyceride (mmol/L)	1.90 ± 0.93	1.56 ± 0.71	1.59 ± 0.84	1.43 ± 0.51	1.79 ± 0.89	1.50 ± 0.62	0.276
HDL‐C (mmol/L)	1.09 ± 0.42	1.03 ± 0.34	1.22 ± 0.25	1.28 ± 0.35	1.13 ± 0.37	1.13 ± 0.35	0.993
LDL‐C (mmol/L)	2.20 ± 0.71	2.11 ± 0.69	1.61 ± 0.62	2.68 ± 1.03	2.00 ± 0.72	2.35 ± 0.86	0.203
Haemoglobin (g/L)	118.50 ± 24.86	115.60 ± 18.01	108.33 ± 10.41	109.14 ± 5.43	115.11 ± 21.35	112.94 ± 14.29	0.728
Albumin (g/L)	41.22 ± 2.31	39.90 ± 2.86	41.68 ± 4.94	39.84 ± 3.51	6.25 ± 1.58	39.87 ± 3.04	0.169
Calcium (mmol/L)	2.27 ± 0.33	2.21 ± 0.18	2.34 ± 0.33	2.19 ± 0.12	2.29 ± 0.32	2.20 ± 0.16	0.271
Phosphorus (mmol/L)	2.00 ± 0.58	1.74 ± 0.27	1.74 ± 0.13	1.92 ± 0.47	1.91 ± 0.49	1.82 ± 0.37	0.525
CRP (mg/L)	1.01 (0.63–3.77)	3.19 (1.85–6.80)	1.98 (0.52–3.24)	1.37 (0.84–2.13)	1.12 (0.60–3.13)	2.18 (0.94–4.94)	0.171
Duration of dialysis (month)	110.50 (61.00–182.50)	114.00 (74.25–140.50)	217.50 (123.00–248.25)	108.00 (68.00–149.00)	126.50 (69.25–214.50)	108.00 (74.00–141.00)	0.391
Kt/V	1.26 ± 0.04	1.27 ± 0.26	1.25 ± 0.04	1.24 ± 0.03	1.25 ± 0.04	1.25 ± 0.03	0.944
Measures of sarcopenia							
HS (kg)	25.61 ± 3.47	25.53 ± 5.74	17.87 ± 3.18	18.89 ± 3.66	23.03 ± 4.99	22.79 ± 5.91	0.900^**a**^
SMI (kg/m^2^)	6.45 ± 0.39	6.42 ± 0.62	5.18 ± 0.39	5.07 ± 0.27	6.03 ± 0.72	5.87 ± 0.84	0.542^**a**^
6‐m gait speed (m/s)	1.04 ± 0.13	1.06 ± 0.13	0.96 ± 0.10	0.97 ± 0.08	1.02 ± 0.12	1.02 ± 0.12	0.890^**a**^

Abbreviations: BMI, body mass index; CON, control group; CRP, C‐reactive protein; EA, electroacupuncture group; HDL‐C, high‐density lipoprotein cholesterol; HS, handgrip strength; Kt/V, fractional clearance index for urea; LDL‐C, low‐density lipoprotein cholesterol; SMI, skeletal muscle index.

^a^ Significant difference between change scores of men and women, *p* < 0.05.

In addition, after the EA treatment, the number of people with sarcopenia in the EA group decreased by six men and three women, and the improvement rates of sarcopenia were 60.00% and 42.86% respectively.

### 3.2 Primary Outcome

#### 3.2.1 6‐M Gait Speed

Compared with the baseline values of their respective groups, the 6‐m gait speed of the EA group increased after receiving EA intervention (Δ = 0.10 ± 0.08; *p* < 0.001), whereas that of the control group decreased (Δ = −0.06 ± 0.09; *p* = 0.018). Repeated measures ANOVA showed that the main effect of time was not significant (*p* = 0.070, η^2^ *p* = 0.096), but the main effect of group was significant (*p* = 0.030, η^2^ *p* = 0.135). The time × group interaction was significant (*p* < 0.001, η^2^ *p* = 0.423), indicating that there was a significant difference in the trend of 6‐m gait speed changes between the EA group and the control group; that is, the improvement effect of EA treatment on 6‐m gait speed was better than that of the control group. The analysis of variance stratified by gender showed that both men (Δ = 0.10 ± 0.08; *p* = 0.003) and women (Δ = 0.11 ± 0.08; *p* = 0.013) in the EA group had an increase in 6‐m gait speed after EA treatment compared to the baseline. There was no difference in the increase amplitude of 6‐m gait speed between the two genders (Table 3).

**TABLE 3:** Change scores (Δ) in primary and secondary outcomes, adjusted for the preintervention values; mean ± SD [95% CI].

	CON‐men (*n* = 12)	EA‐men (*n* = 10)	CON‐women (*n* = 6)	EA‐women (*n* = 7)	CON (*n* = 18)	EA (*n* = 17)
Primary outcome						
6‐m gait speed (m/s)						
Baseline (mean ± SD)	1.04 ± 0.13	1.06 ± 0.13	0.96 ± 0.10	0.97 ± 0.08	1.02 ± 0.12	1.02 ± 0.12
8 weeks (mean ± SD)	0.98 ± 0.09	1.16 ± 0.14	0.92 ± 0.08	1.08 ± 0.12	0.96 ± 0.09	1.12 ± 0.3
Δ (post–pre)	−0.06 ± 0.08^b^, ^c^ [−0.11 to −0.02]	0.10 ± 0.08^b^, ^c^ [0.05–0.15]	−0.04 ± 0.11^c^ [−0.13–0.05]	0.11 ± 0.08^b^, ^c^ [0.03–0.19]	−0.06 ± 0.09^b^, ^c^ [−0.09 to −0.02]	0.10 ± 0.08^b^, ^c^ [0.07–0.14]
Secondary outcome						
HS, (kg)						
Baseline (mean ± SD)	25.61 ± 3.47	25.53 ± 5.74	17.87 ± 3.18	18.89 ± 3.66	23.03 ± 4.99	22.79 ± 5.91
8 weeks (mean ± SD)	25.24 ± 3.64	26.47 ± 5.63	16.32 ± 3.70	19.20 ± 3.97	22.27 ± 5.60	23.48 ± 6.11
Δ (post–pre)	−0.37 ± 1.05^a^, ^c^ [−1.02–0.29]	0.94 ± 1.08^b^, ^c^ [0.22–1.66]	−1.50 ± 1.11^a^, ^b^, ^c^ [−2.35 to −0.66]	0.31 ± 0.75^c^ [−0.51–1.06]	−0.76 ± 1.19^b^, ^c^ [−1.29 to −0.30]	0.68 ± 0.98^b^, ^c^ [0.21–1.23]
SMI (kg/m^2^)						
Baseline (mean ± SD)	6.45 ± 0.39	6.42 ± 0.62	5.18 ± 0.39	5.07 ± 0.27	6.03 ± 0.72	5.87 ± 0.84
8 weeks (mean ± SD)	6.43 ± 0.48	6.60 ± 0.61	5.25 ± 0.51	5.27 ± 0.44	6.03 ± 0.74	6.05 ± 0.86
Δ (post–pre)	−0.03 ± 0.21^c^ [−0.16–0.11]	0.18 ± 0.21^b^, ^c^ [0.04–0.32]	0.07 ± 1.97 [−0.15–0.24]	0.20 ± 0.24 [0.04–0.40]	0.01 ± 0.20^c^ [−0.10–0.11]	0.19 ± 0.22^b^, ^c^ [0.08–0.30]
Sarcopenia, *n* (%)	11	4	4	4	15	8

Abbreviations: CON, control group; EA, electroacupuncture group.

^a^ Significant difference between change scores of men and women, *p* < 0.05.

^b^ Significant difference compared with baseline, *p* < 0.05.

^c^ Significant group × time interaction, *p* < 0.05.

### 3.3 Secondary Outcomes

#### 3.3.1 Skeletal Muscle Index (SMI)

Compared with their respective baselines, the SMI in the EA group increased after EA treatment (Δ = 0.19 ± 0.22; *p* = 0.003), although there was no significant change in the control group. Repeated measures ANOVA showed that the main effect of time on SMI was significant (*p* = 0.011, η^2^ *p* = 0.181), and the main effect of group was not significant (*p* = 0.788, η^2^ *p* = 0.002). Moreover, the time × group interaction was significant (*p* = 0.016, η^2^ *p* = 0.164), indicating that the trends of SMI changes over time in the EA group and the control group were significantly different. The EA treatment was more effective than the control group in maintaining SMI. The data stratified by gender showed that compared with their respective control groups, the SMI of women in the EA group significantly increased (Δ = 0.18 ± 0.21; *p* = 0.027) (Table 3).

#### 3.3.2 Handgrip Strength

Compared with their respective baseline values, handgrip strength in the EA group increased after EA treatment (Δ = 0.68 ± 0.98; *p* = 0.011), whereas that in the control group decreased (Δ = −0.76 ± 1.19; *p* = 0.015). Analysis showed that neither the main effect of time (*p* = 0.561, η^2^ *p* = 0.010) nor the main effect of group (*p* = 0.853, η^2^ *p* = 0.001) was significant; however, the time × group interaction was significant (*p* = 0.002, η^2^ *p* = 0.264), indicating a significant difference in the trend of handgrip strength changes between the EA group and the control group. The improving effect of EA treatment on handgrip strength was superior to that of the control group. The analysis of variance stratified by gender showed that compared with their respective control groups, the men in the EA group showed an increase (Δ = 0.94 ± 1.08, *p* = 0.022), whereas the women in the control group showed a decrease (Δ = −1.50 ± 1.11; *p* = 0.019) (Table 3).

### 3.4 Adverse Events

No serious side effects associated with EA treatment were reported. During the entire trial process, there were no cases of fainting, subcutaneous hematoma, local infection, significant pain, allergy or other systemic adverse reactions. The most commonly reported adverse event was slight oozing of blood beads when the patient withdrew the needle at the end of EA treatment. This was a mild and common local physiological reaction of EA treatment, and it was relieved immediately after using sterile cotton swabs for care.

### 3.5 Metabolomics Analysis of Serum in the EA Group

**FIGURE 3:**
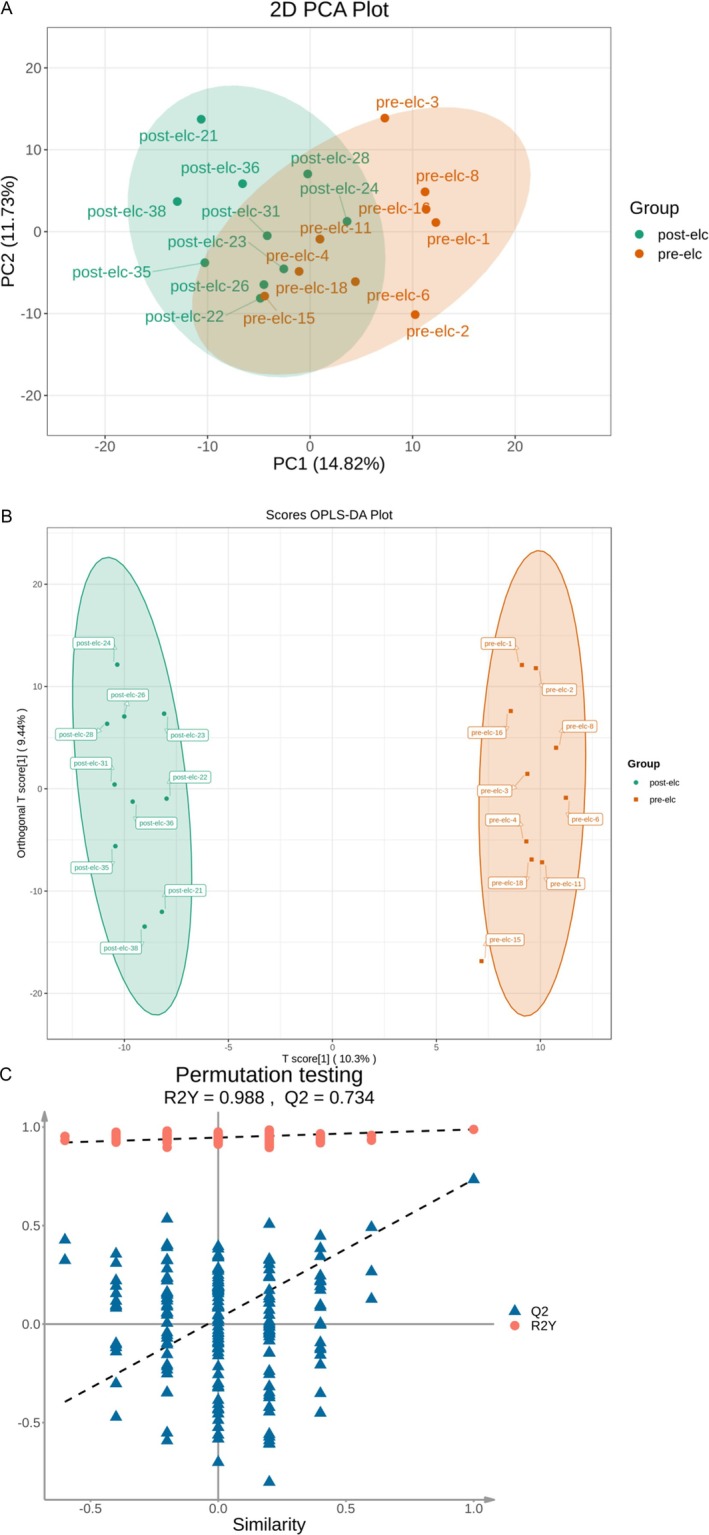
(A) Results of principal component analysis (PCA). Pre‐ele: The sample group before the EA group received the treatment. Post‐ele: The sample group after the EA group received the treatment. (B) Results of orthogonal partial least squares discriminant analysis (OPLS‐DA). Pre‐ele: the sample group before the EA group received the treatment. Post‐ele: The sample group after the EA group received the treatment. (C) Results of the permutation test (*n* = 200).

After obtaining the metabolomic analysis data, principal component analysis was performed on the baseline and post‐treatment samples of the EA group. PCA and OPLS‐DA were utilized to model and assess the systemic changes in metabolism within the EA group. The findings revealed a distinct separation of metabolic features, suggesting that the plasma metabolic profile of the patients underwent significant changes post‐EA treatment compared to pre‐EA treatment (Figure 3A,B). The OPLS‐DA model parameters for this comparison were R^2^Y = 0.988 and Q^2^ = 0.734. Based on the permutation test results (*n* = 200), no evidence of data overfitting was detected (Figure 3C).

**FIGURE 4:**
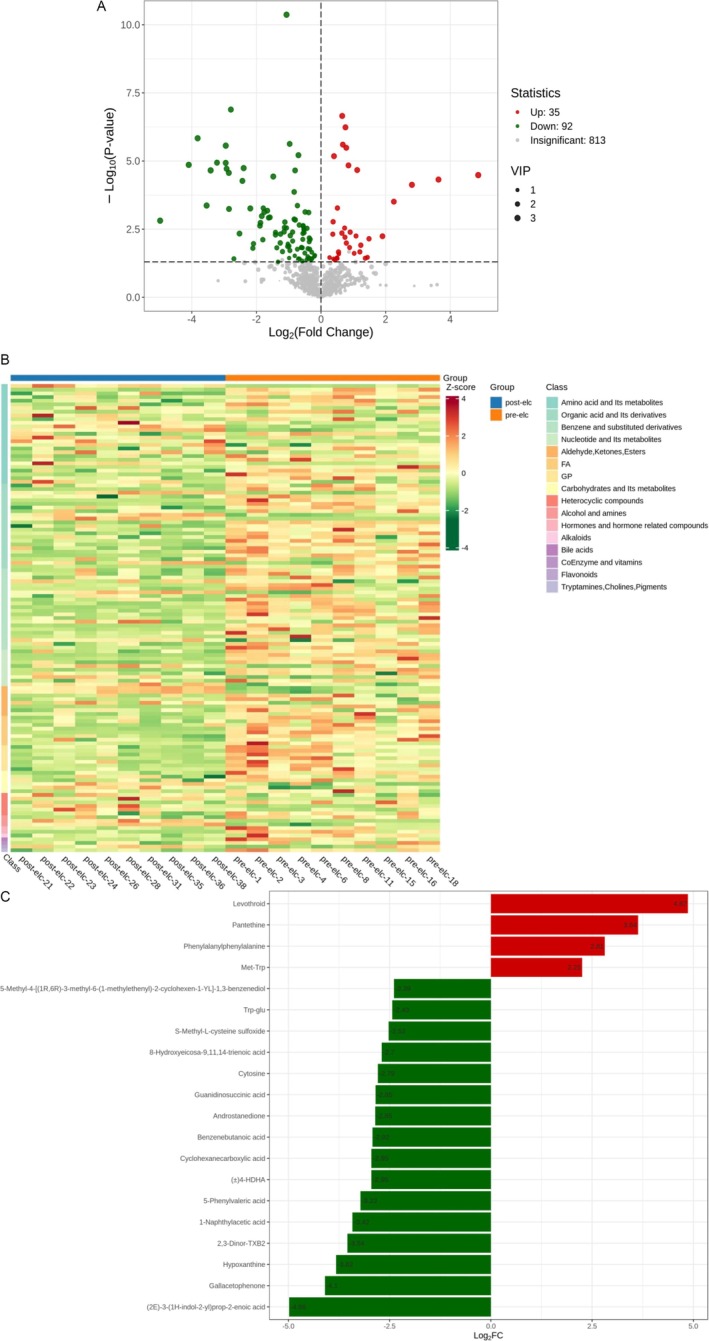
(A) Volcano plot of differential metabolites. (B) Heat map of differential metabolite clustering. Pre‐ele: The sample group before the EA group received the treatment. Post‐ele: The sample group after the EA group received the treatment. (C) Fold difference bar for the top 20 ranked substances. Red represents upregulation of metabolite content and green represents downregulation of metabolite content.

**FIGURE 5:**
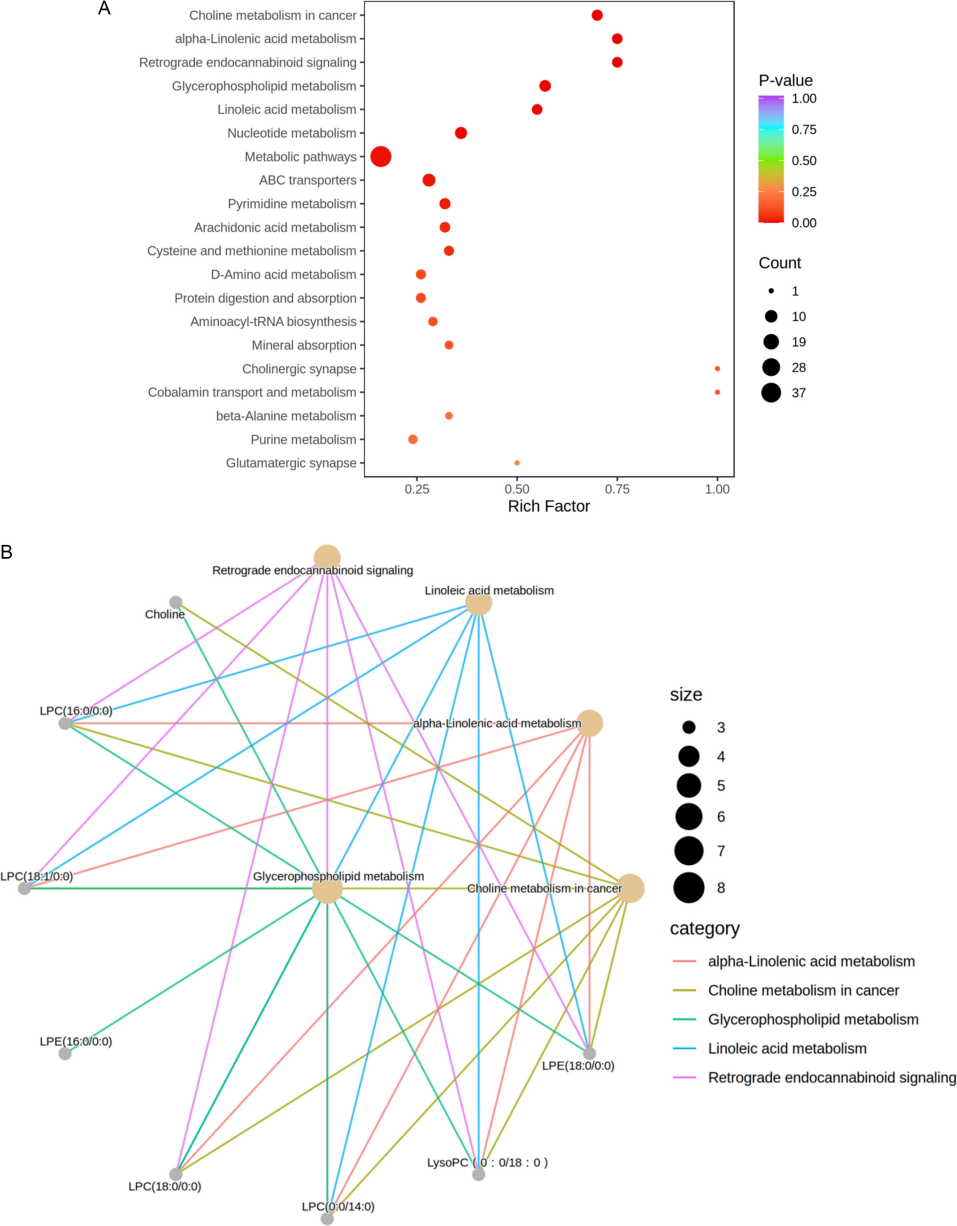
(A) Bubble map of KEGG pathway enrichment. (B) Enriched cnet network diagram.

Following data integration, a total of 127 differential metabolites were enriched and identified (*p* < 0.05, VIP > 1), comprising 35 up‐regulated metabolites and 92 down‐regulated metabolites (Figure 4A). These metabolites are primarily amino acids, organic acids, benzene and its substituted derivatives, nucleotides and fatty acids (Figure 4B). Among them, the 20 metabolites with the highest difference in fold change are shown in Figure 4C. Building upon the differential metabolites identified previously, we explored the potential metabolic pathways through which EA treatment might treat sarcopenia in patients undergoing MHD. Our analyses utilized KEGG metabolic pathway enrichment and were visualized using bubble plots. The findings indicated that the five most enriched pathways were glycerophospholipid metabolism, linoleic acid metabolism, alpha‐linolenic acid metabolism, retrograde endocannabinoid signalling and choline metabolism in cancer (Figure 5A,B).

## 4 Discussion

The results demonstrated that compared with the control group, EA treatment could lead to higher 6‐m gait speed, handgrip strength and SMI in sarcopenia patients undergoing MHD. During the EA treatment, no serious adverse reactions were observed. This indicated that EA can effectively treat sarcopenia patients undergoing MHD. In addition, serum metabolomics results indicated that the patients' metabolism changed after EA treatment, which may be closely related to the positive regulation of systemic metabolism (including amino acid and lipid profiles).

In patients undergoing MHD, the incidence of sarcopenia is particularly prominent, mainly caused by age and the dialysis process [26]. A variety of related diseases contribute to this, including protein loss, a micro‐inflammatory state, metabolic acidosis and insulin resistance [27, 28]. In recent years, treatment methods for sarcopenia have been continuously updated [29]. However, there remains a lack of a safe and effective treatment specifically for patients undergoing MHD [30]. Several previous studies have shown that acupuncture treatment in patients undergoing MHD can improve symptoms such as fatigue, stress, hypertension and pruritus [31, 32]. Hu et al. [33] conducted a fundamental experiment study demonstrating that low‐frequency electrical stimulation ameliorated skeletal muscle atrophy in chronic kidney disease by up‐regulating IGF‐1 signalling, thereby enhancing protein metabolism and promoting myogenesis. Furthermore, an animal study by Guo et al. [34] indicated that EA treatment combined with sulforaphane could repair mitochondrial damage, thereby improving the morphology and function of skeletal muscles. It is worth noting that there are few studies on the efficacy and mechanism of acupuncture in treating sarcopenia, and the evaluations vary, with most being limited to basic research.

In this clinical cohort study, the results showed that the 6‐m gait speed in the EA group significantly increased. Previous research has demonstrated that gait speed is a reliable indicator of sarcopenia and a powerful predictor of mortality [35]. The improvement in 6‐m gait speed was observed in both men and women participants. The handgrip strength of the EA group significantly increased. Through gender‐stratified analysis, it was found that the improvement in handgrip strength was mainly attributed to the men participants. It should be noted that the between‐group difference in handgrip strength (approximately 1.44 kg, *p* < 0.01) did not reach the 5–6.5 kg threshold of the minimal clinically important difference (MCID) reported for non‐CKD patients with sarcopenia [36]. However, given that low handgrip strength is an independent predictor of death in MHD patients [37, 38], we believe that this improvement holds clinical significance. In addition to functional indicators, EA treatment also had a positive impact on muscle mass, manifested as a significant increase in the SMI in the EA group. Similar to the handgrip strength results, the increase in SMI was more significant in men. In conclusion, these results strongly indicated that EA treatment was a promising therapeutic measure for sarcopenia. Furthermore, on the basis of previous sufficient investigation, this study conducted a small‐scale pre‐experiment of EA treatment on eligible patients. This clinical study was carried out under the condition of the safety and success of the pilot study. It is innovatively proposed to perform EA on both lower limbs during stable dialysis. This treatment can not only improve the sarcopenia of patients undergoing MHD but also save patients' time and help to improve the quality of life, which has great clinical application value.

In the serum metabolomics analysis, we observed significant changes in serum metabolites following EA treatment. Among these, amino acids, organic acids, benzene and its substituted derivatives, nucleotides and fatty acids exhibited significant alterations. Further analysis indicated that the pathways in which EA treatment might participate in treating sarcopenia primarily involve lipid‐related metabolic pathways. It is worth noting that the clinical improvements in physical function and muscle mass achieved after EA treatment can be reasonably explained at the level of our serum metabolomics findings. The significant changes in amino acids, fatty acids and energy‐related metabolites observed collectively suggest a systemic metabolic remodelling. Specifically, the changes in amino acids may directly support the increase in SMI by promoting a more favourable metabolic environment conducive to muscle protein synthesis. At the same time, the optimization of lipid and energy metabolism patterns reflected in the identified lipid‐related pathways is likely to improve the efficiency of muscle energy utilization. This metabolic transformation can provide the necessary biological energy substrates for continuous muscle contraction, thereby manifesting as the improvement in gait speed and handgrip strength that we observed. Additionally, the reduction of uraemic toxin precursors is particularly important for patients undergoing MHD, as it may improve the chronic inflammatory and catabolic environment that existed in their bodies [39, 40].

To sum up, we boldly hypothesize that EA treatment does not act through a single mechanism but coordinates a multifaceted recovery process. It may counteract uraemic metabolic disorders, reprogramme energy metabolism to favour muscle performance and create a favourable metabolic environment for muscle mass growth and functional enhancement. Although the precise causal chain needs further verification, the strong consistency between phenotypic recovery and systemic metabolic remodelling strongly indicates that the improvement of sarcopenia in patients undergoing MHD is intrinsically associated with this EA‐induced metabolic homeostasis. To our knowledge, this study is the first to examine the efficacy of EA treatment in sarcopenia patients undergoing MHD, as well as to address serum metabolomics. We sincerely hope that our findings can aid in guiding appropriate treatment decisions for MHD patients with sarcopenia and promote the clinical application of EA treatment in the management of sarcopenia.

Despite the efforts, several limitations should be acknowledged. First, blinding of participants and therapists was not feasible. Second, the sample size was small and from a single centre; thus, multicentre studies with larger samples are needed. Third, we could not obtain skeletal muscle tissue from patients for metabolomics analysis due to ethical considerations; subsequent basic research will address this. Fourth, unavoidable clinical confounders (e.g., daily medications) may have influenced metabolomic results, requiring further validation through basic experiments. Fifth, patients with severe conditions or unable to walk independently were excluded, limiting generalizability to those with advanced sarcopenia. Sixth, at the study design stage, no EA/acupuncture RCTs in MHD patients were available; therefore, sample size calculation relied on reported 6‐min walk distance data. Future studies should establish population‐specific reliability and effect size for gait speed. Seventh, EA was applied only to lower limb acupoints; the mechanism behind improved handgrip strength remains unclear, possibly involving systemic indirect effects (e.g., reducing inflammation or enhancing lower limb activity). Further research is needed to clarify this.

In conclusion, the evidence provided by this study indicates that EA treatment may serve as a beneficial intervention for improving sarcopenia in patients undergoing MHD. We observed meaningful improvements in patients' gait speed, handgrip strength and SMI after EA treatment. The accompanying serum metabolomics analysis suggested that these improvements might be related to the positive regulation of systemic metabolism (including amino acid and lipid profiles). Moreover, this therapy can be implemented during haemodialysis, which highlights its practicality and potential as a feasible clinical method and merits further investigation in larger clinical trials.

## Funding

This study was supported by the Jiangsu Province Project for the Development of Traditional Chinese Medicine Science and Technology (grant number MS2025129), the National Natural Science Foundation of China (grant number 82572522), the Key Scientific Research Project of Integrated Traditional Chinese and Western Medicine at Zhongda Hospital affiliated with Southeast University (grant number 2023zxyxt01) and the Social Development Project in Hailing District of Taizhou City (grant number HLKF‐2024‐5).

## Ethics Statement

This study was approved by the IEC for clinical research of Zhongda Hospital Affiliated to Southeast University (batch number: 2023ZDSYLL371‐P01). The trial has been registered in the Chinese Clinical Trial Registry (registration number: ChiCTR2400081511). The research was conducted in accordance with the principles of the Declaration of Helsinki. Informed consent for the study was obtained from all subjects and/or their legal guardians by signing an informed consent form.

## Conflicts of Interest

The authors declare no conflicts of interest.

## Supporting information


**Data S1:** Supporting Information.

## Data Availability

The data analysed in the current study are available from the corresponding author on reasonable request.
