# Supplementary material for: Electroacupuncture Treatment on Sarcopenia in Patients Undergoing Maintenance Haemodialysis: An Effective Therapy
Source: J Cachexia Sarcopenia Muscle. 2026 Jul 19;17(4):e70345. doi: 10.1002/jcsm.70345 (PMC13381724; doi:10.1002/jcsm.70345)
Supplement: Supplementary file 1 — Data S1: Supporting Information. [file JCSM-17-e70345-s001.docx]

**Supplementary materials**

**Metabolomics study**

**Sample preparation and extraction**

Fasting cubital venous blood samples were collected from all participants at baseline and at the end of the experiment (week 8) for metabolomics analysis. All collected blood samples were immediately sent to the Biobank of Zhongda Hospital affiliated with Southeast University, for centrifugation. 50μL of sample and 300μL of extraction solution (ACN:Methanol=1:4, V/V) containing internal standards were added into a 2mL microcentrifugetube. The sample was vortexed for 3 min and then centrifuged at 12000rpm for 10 min (4°C). 200 μL of the supernatant was collected and placed in -20°C for 30 min, and then centrifuged at 12000rpm for 3 min (4°C). A 180μL aliquots of supernatant were transferred for LC-MS analysis.

**Experimental procedure**

All samples were for two liquid chromatography-mass spectrometry (LC-MS) methods. One aliquot was analyzed using positive ion conditions and was eluted from T3 column (Waters ACQUITY Premier HSS T3 Column 1.8 µm, 2.1 mm * 100 mm) using 0.1% formic acid in water as solvent A and 0.1% formic acid in acetonitrile as solvent B. Elution was carried out according to a specific gradient (0-2 minutes, 5%-20%B; 2-5 minutes, 20%-60%B; 5-6 minutes, 60%-99%B; 6-7.5 minutes, 99%A; 7.5-7.6 minutes, 99%-5%A, 7.6-10 minutes, 5%A). The analytical conditions were as follows, column temperature, 40 °C; flow rate, 0.4 mL/min; injection volume, 4 μL; Another aliquot was using negative ion conditions and was the same as the elution gradient of positive mode. The data acquisition was operated using the information-dependent acquisition (IDA) mode using Analyst TF 1.7.1 Software. Two ionization modes, positive ions and negative ions, were adopted. The key source parameters were set as follows: ion source temperature: 550°C; ion spray voltage: ±5000 volts (positive/negative mode); dissociation potential: ±60 volts.

**Analytical method**

The raw data obtained from the mass spectrometry was converted to MZML format using ProteoWizard. Peak extraction, alignment, and retention time correction were performed using the XCMS program. The peaks after correction were identified as metabolites by searching the laboratory's self-built database, integrating public libraries, prediction libraries, and the metDNA method. The search results were scored based on the search errors of the parent ion Q1 (25 ppm), MS2 (50 ppm), and retention time (60 s). The databases include: Metlin (http://metlin.scripps.edu/index.php), HMDB 4.0 (https://hmdb.ca/), KEGG (https://www.kegg.jp/), Mona (https://mona.fiehnlab.ucdavis.edu/), MassBank (http://www.massbank.jp/); prediction libraries, based on MetDNA and AI libraries.

Unsupervised Principal Component Analysis (PCA) was performed using the statistical function prcomp in R language (website: www.r-project.org). For the two groups of analysis, VIP (VIP>1) and *P* values (*P*<0.05, Student's t-test) were extracted from the orthogonal partial least squares discriminant analysis (OPLS-DA) results to determine the differential metabolites. The data was log transform (log2) and mean centering before OPLS-DA. In order to avoid overfitting, a permutation test (200 permutations) was performed. Identified metabolites were annotated using kyoto encyclopedia of genes and genomes (KEGG) Compound database (http://www.kegg.jp/kegg/compound/), annotated metabolites were then mapped to KEGG Pathway database (http://www.kegg.jp/kegg/pathway.html). The results of serum metabolomics were performed using the Metware Cloud, an online platform for data analysis ( https://cloud.metware.cn ).
